# High variability of Blue Carbon storage in seagrass meadows at the estuary scale

**DOI:** 10.1038/s41598-020-62639-y

**Published:** 2020-04-03

**Authors:** Aurora M. Ricart, Paul H. York, Catherine V. Bryant, Michael A. Rasheed, Daniel Ierodiaconou, Peter I. Macreadie

**Affiliations:** 1 Bodega Marine Laboratory- Department of Earth and Planetary Sciences, University of California Davis, Davis, USA; 2 Departament de Biologia Evolutiva, Ecologia i Ciències ambientals, Universitat de Barcelona, Barcelona, Spain; 3 Centre for Tropical Water and Aquatic Ecosystem Research, James Cook University, Townsville, Australia; 4 School of Life and Environmental Sciences, Centre for Integrative Ecology, Deakin University, Burwood, Australia

**Keywords:** Marine biology, Carbon cycle, Climate-change mitigation, Ecosystem services

## Abstract

Seagrass meadows are considered important natural carbon sinks due to their capacity to store organic carbon (C_org_) in sediments. However, the spatial heterogeneity of carbon storage in seagrass sediments needs to be better understood to improve accuracy of Blue Carbon assessments, particularly when strong gradients are present. We performed an intensive coring study within a sub-tropical estuary to assess the spatial variability in sedimentary C_org_ associated with seagrasses, and to identify the key factors promoting this variability. We found a strong spatial pattern within the estuary, from 52.16 mg C_org_ cm^−3^ in seagrass meadows in the upper parts, declining to 1.06 mg C_org_ cm^−3^ in seagrass meadows at the estuary mouth, despite a general gradient of increasing seagrass cover and seagrass habitat extent in the opposite direction. The sedimentary C_org_ underneath seagrass meadows came principally from allochthonous (non-seagrass) sources (~70–90 %), while the contribution of seagrasses was low (~10–30 %) throughout the entire estuary. Our results showed that C_org_ stored in sediments of seagrass meadows can be highly variable within an estuary, attributed largely to accumulation of fine sediments and inputs of allochthonous sources. Local features and the existence of spatial gradients must be considered in Blue Carbon estimates in coastal ecosystems.

## Introduction

Seagrass ecosystems are among the most significant natural carbon sinks worldwide, since they can sequester significant amounts of carbon, store it as organic carbon (C_org_) in the sediments for long periods of time, and have a worldwide distribution^1–3^. It is estimated that seagrass ecosystems store globally up to 19.9 petagrams (Pg) of C_org_ in sediments^2^, or between 4.2 and 8.4 Pg of C_org_ from a more conservative approach^2^, where C_org_ could be stored in sediments for hundreds of years and even millennia^4^.

Due to the high mitigation potential for seagrass ecosystems to help offset carbon dioxide (CO_2_) emissions, there has been a major effort in recent years to improve the accuracy of estimates of carbon stored in seagrass sediments, and so, include seagrass ecosystems within greenhouse gases (GHG) abatement schemes^5,6^. Consequently, an increasing number of studies have attempted to quantify sedimentary C_org_ stocks associated with seagrass meadows from direct measurements^7–9^, demonstrating an enormous variability in the seagrass sedimentary C_org_ stocks reported worldwide.

Variability of sedimentary C_org_ stocks has been associated with multiple interrelated biological and environmental factors: type of seagrass species^7^, depth and light availability^10,11^, landscape configuration^12^, physical disturbances^13^, wave height and turbidity^14^, and even faunal presence such as bioturbators and top predators^15,16^ Most of these studies highlighted the type of carbon sources and the sediment grain size as the main factors influencing carbon storage in seagrass sediments, suggesting that the processes affecting these factors explain the high variability found in seagrass sedimentary C_org_ stocks^17^.

Variability in seagrass sedimentary C_org_ stocks has been described at a range of spatial scales: from cm and meters, at the seagrass patch and meadow scale^18–20^ to bioregions and latitudinal scales^7,9^. Studies at small scales (up to ~1′s km) have shown how sedimentary C_org_ can be spatially distributed inside seagrass meadows^18,20,21^. However, in studies at higher spatial scales (~100′s to ~1000′s km), comparisons and estimates are usually made based on a relatively low number of sediment cores from each site^22–24^, therefore, not accounting for the potential spatial variability in seagrass sedimentary C_org_ stocks among meadows in the same area. Just a few studies focus on the variability at intermediate spatial scales (~10′s km), comparing among seagrass meadows within a local system^14,25,26^, such as estuaries, where seagrasses can make up a large proportion of available habitat^27^.

Estuaries are dynamic transition zones acting as pathways for the transfer of sediments and organic materials from land to the sea. Estuaries are characterized by a high variability in geo-morphology, and the presence of spatial gradients, influenced by large tidal cycles, longer water residence times and poorer mixing compared to the open ocean. Carbon fluxes to estuarine sediments can be conceptualized in two main axes^28^. The horizontal axis is determined by a hydrodynamic control, exerted between the river and the ocean and/or following estuary geo-morphology, where terrestrial carbon is directly delivered and rapidly deposited in the sediments^29^, thus creating a strong spatial gradient of C_org_ in sediments from the upper parts of the estuary to the ocean. The vertical axis is determined by a biological control, where C_org_ in sediments is the result of direct deposition and burial of *in situ* photosynthetic production between the surface (i.e. phytoplankton) and the benthos (i.e. seagrasses and/or algae if present). The prevalence of one control or the other will largely determine the magnitude and origin of C_org_ stocks in the sediments within estuaries, and also its spatial distribution, thus, expecting a more homogeneous spatial distribution of C_org_ in sediments of seagrass meadows with a prevalence of biological control, and a more heterogeneous distribution where hydrodynamic control dominates.

Variation also exists among and within estuaries, in terms of turbidity and water flow, which may determine seagrass meadows’ habitat extent or landscape configuration^30,31^ and also species composition and structural traits, such as shoot density and cover^32^. Other coastal vegetated habitats, such as mangroves and saltmarshes can be present in estuaries also contributing to C_org_ stocks^33,34^ and to seagrass sedimentary C_org_ stocks^35^. Variation is also reflected in sedimentation rates, which can be influenced by seagrass canopies trapping particulate organic materials, thus decreasing turbidity and increasing sedimentary C_org_ stocks^36^. Some of these factors may determine spatial patterns of C_org_ storage in sediments of seagrass meadows. However, despite the large number of studies that have appeared recently providing empirical evidence of processes explaining C_org_ storage in seagrass sediments, there are a lack of studies providing the spatial component needed to improve accuracy of Blue Carbon assessments, particularly at the within estuary scale. The current work aimed to contribute to understanding the spatial variability of C_org_ storage in seagrass sediments by providing a comprehensive assessment of the spatial distribution of sedimentary C_org_ stocks and carbon sources in seagrass meadows within a coastal plain estuary and exploring the site-specific factors driving the patterns found.

We expected that sedimentary C_org_ storage in seagrass meadows within the estuary will present a high spatial heterogeneity due to the presence of spatial gradients of environmental and biological factors. Therefore, we investigated how sediment C_org_ content (%), C_org_ stocks (mg C_org_ cm^−3^) and carbon sources (δ^13^C) vary as a function of seagrass cover, meadow extent or landscape configuration (hereinafter “meadow type”), sediment particle size, water turbidity and the proximity to mangroves, as the main environmental and biological factors present within the estuary studied that have been already described to be affecting C_org_ storage in seagrass sediments^12,14,26^. Specifically, we hypothesized that (1) seagrass sedimentary C_org_ storage will increase with seagrass cover and will be higher in continuous meadows due to a greater accumulation of seagrass carbon sources and fine sediments; (2) proximity to mangroves and a higher water turbidity will increase sedimentary C_org_ storage due to higher contribution of allochthonous carbon sources.

## Results

All sediment parameters and environmental and biological drivers are summarized in Table 1. The sediment C_org_ content in seagrass meadows within the estuary ranged from 0.08% to 6.01%, and C_org_ stocks ranged from 1.06 mg C_org_ cm^−3^ to 52.16 mg C_org_ cm^−3^, where in both cases the lowest values were found in seagrass meadows at the mouth of the estuary and the highest values in seagrass meadows at the upper estuary reaches. Values of δ^13^C were in general highly depleted along the estuary, from −18.32‰ to −26.20‰, with enriched (more positive) values in sediments of seagrass meadows at the mouth of the estuary and more depleted values in sediments in the upper estuary. Similar patterns were found at all depths analysed. All three sediment carbon variables assessed presented spatial autocorrelation on the original data (Moran I test p < 0.01; Supplementary Table S1). Sediment dry bulk density ranged from 0.38 g cm^−3^ to 3.08 g cm^−3^, with the lowest values in seagrass meadows at the upper parts of the estuary and the highest in seagrass meadows at the mouth. The proportion of fine sediments (<63 µm) ranged from 3.16% to 93.92%, with the lowest values in seagrass meadows at the mouth of the estuary and the highest in seagrass meadows at the upper estuary.

**Table 1:** Environmental and biological drivers, and sediment parameters.

Site	Environmental and Biological drivers					Sediment parameters									
	Seagrass species and number of cores sampled in brackets	Seagrass cover (%)	Meadow type	Turbidity level	Distance to mangroves (m)	<63 µm (%)		DBD (g cm^−3^)		C_org_ (%)	δ^13^C (‰)		C_org_ stocks (mg C_org_ cm^−3^)		
Redcliffe	*Z. muelleri (1); H. ovalis (1); H. decipiens (1)*	0–1	patchy	high	140–140	80.17	85.19	0.38	0.87	2.81 6.01	−26.00	−24.46	12.91	52.16	
Black Swan	*Z. muelleri (3)*	0–10	patchy	high	200–300	27.82	75.92	0.86	1.48	0.87 2.92	−25.67	−24.72	11.26	35.58	
Fishermans Landing	*Z. muelleri (2); H. ovalis (1)*	0–1	patchy	medium	515–565	47.16	85.47	0.82	1.47	0.44 2.51	−25.37	−23.32	4.60	25.11	
Channel Islands	*Z. muelleri (3)*	5–20	patchy	medium	100–200	8.29	35.60	1.25	2.97	0.28 2.03	−25.09	−22.51	4.55	31.81	
Wiggins Island	*Z. muelleri (3)*	0–10	patchy	high	610–680	50.62	73.73	0.90	1.45	0.19 0.99	−24.10	−22.99	2.08	10.79	
Grahams Creek	*H. decipiens (3)*	0–5	patchy	high	35–35	56.37	93.92	0.71	1.04	1.50 4.55	−26.20	−25.29	11.61	33.84	
Pelican Banks South 1	*Z. muelleri (3)*	0–15	continuous	medium	3500–3500	9.49	12.47	1.38	2.12	0.39 1.45	−22.07	−20.90	7.07	21.62	
Pelican Banks South 2	*Z. muelleri (2); H. ovalis (1)*	10–20	continuous	medium	3400–3400	7.73	11.56	1.37	1.93	0.37 2.08	−21.83	−19.11	5.21	34.57	
Pelican Banks North 1	*Z. muelleri (3)*	30–65	continuous	low	1230–1230	13.69	16.55	1.13	1.83	0.44 1.03	−19.99	−18.51	5.96	17.15	
Pelican Banks North 2	*Z. muelleri (3)*	45–70	continuous	low	1120–1120	15.02	18.43	1.06	1.63	0.39 1.17	−19.44	−18.61	5.65	12.41	
Facing Island	*Z. muelleri (2); H. ovalis (1)*	0–5	patchy	medium	3255–3280	7.45	28.87	1.25	1.91	0.24 1.42	−23.35	−19.87	4.47	19.67	
Pelican Banks North 3	*Z. muelleri (3)*	57–60	continuous	low	1950–1950	9.91	16.11	1.33	1.75	0.3 1.72	−19.17	−18.32	4.70	24.86	
South Trees	*Z. muelleri (3)*	0–1	variable	medium	150–250	5.96	17.71	1.12	1.95	0.18 1.57	−24.11	−20.44	3.49	17.52	
Boyne Island	*H. uninervis (3)*	2–10	variable	medium	1000–1025	3.16	3.45	1.07	3.08	0.08 0.25	−22.89	−20.43	1.06	3.68	
South Facing Island	*Z. muelleri (3)*	0–10	variable	medium	250–250	12.50	17.35	1.45	2.54	0.09 1.41	−21.99	−20.40	1.30	22.18	

A total of 45 cores were collected in 15 sites along the estuary. For summary purposes data is shown per site but cores are treated independently on the spatial GLS models applied. Data show ranges of minimum and maximum values or category of each variable.

The non-spatial GLS models (except those with meadow type as explanatory variable and those assessing C_org_ (%) with proportion of fine sediments) presented spatial autocorrelation on the residuals (Moran I p < 0.01, Supplementary Table S2), showing that the explanatory variables were not removing the effect of spatial dependence among samples, and that error correlation structures were then necessary. All the spatial GLS models, except some cases assessing δ^13^C in core section 1–3 cm, did not present spatial autocorrelation in the residuals (Moran I p > 0.01, Supplementary Table S2). We will therefore describe just the results from spatial GLS models when spatial autocorrelation was removed by the correlation structure (Table 2).

**Table 2:** Coefficient estimates for carbon sediment variables in the GLS spatial models.

Depth	Dependent variable	C_org_ (%)			C stocks (mg C_org_ cm^−3^)			δ^13^C (‰)		
	Driver	∆AIC	Estimate	SE	∆AIC	Estimate	SE	∆AIC	Estimate	SE
0–1 cm	Seagrass cover*	38.75	0.00	0.00	30.61	0.00	0.00	**63.93**	**0.03**	**0.01**
	<63 µm*	**12.28**	**0.01**	**0.00**	18.44	0.00	0.00	47.21	−0.01	0.01
	Distance from mangroves*	36.74	0.00	0.00	33.64	0.00	0.00	71.45	0.00	0.00
	Turbidity (low)	22.78	0.55	0.34	19.26	2.23	0.64	**36.68**	**−20.14**	**0.83**
	Turbidity (medium)	22.78	−0.20	0.27	19.26	−0.12	0.29	**36.68**	**−2.09**	**0.42**
	Turbidity (high)	22.78	0.42	0.39	19.26	0.16	0.40	**36.68**	**−4.05**	**0.57**
	Meadow type (continuous)	24.38	0.51	0.47	**5.34**	**2.52**	**0.17**	**24.64**	**−19.94**	**0.96**
	Meadow type (patchy)	24.38	0.42	0.55	**5.34**	**0.20**	**0.24**	**24.64**	**−4.55**	**1.11**
	Meadow type (variable)	24.38	−0.19	0.56	**5.34**	**−0.90**	**0.27**	**24.64**	**−1.12**	**1.24**
1–3 cm	Seagrass cover*	28.51	0.00	0.00	7.64	0.00	0.01	**61.09**	**0.04**	**0.01**
	<63 µm*	**8.06**	**0.01**	**0.00**	3.21	0.01	0.00	48.06	0.00	0.01
	Distance from mangroves*	30.54	0.00	0.00	8.57	0.00	0.00	60.62	0.00	0.00
	Turbidity (low)	17.52	0.34	0.32	9.27	1.61	1.29	**29.26**	**−19.11**	**0.76**
	Turbidity (medium)	17.52	0.12	0.27	9.27	0.44	0.40	**29.26**	**−2.95**	**0.87**
	Turbidity (high)	17.52	0.67	0.38	9.27	0.50	0.51	**29.26**	**−5.93**	**0.95**
	Meadow type (continuous)	19.21	0.46	0.43	**2.87**	**2.47**	**0.20**	**21.41**	**−19.95**	**0.46**
	Meadow type (patchy)	19.21	0.50	0.50	**2.87**	**0.21**	**0.27**	**21.41**	**−4.71**	**0.62**
	Meadow type (variable)	19.21	−0.19	0.52	**2.87**	**−0.65**	**0.31**	**21.41**	**−1.67**	**0.74**
3–10 cm	Seagrass cover*	26.84	−0.01	0.00	13.19	−0.01	0.01	**48.87**	**0.04**	**0.01**
	<63 µm*	**8.94**	**0.01**	**0.00**	9.92	0.00	0.00	55.64	0.00	0.01
	Distance from mangroves*	27.19	0.00	0.00	11.85	0.00	0.00	69.80	0.00	0.00
	Turbidity (low)	16.28	0.38	0.31	13.04	1.62	1.53	**31.19**	**−18.97**	**1.10**
	Turbidity (medium)	16.28	0.19	0.27	13.04	0.63	0.48	**31.19**	**−3.74**	**1.15**
	Turbidity (high)	16.28	0.56	0.37	13.04	0.38	0.61	**31.19**	**−5.95**	**1.29**
	Meadow type (continuous)	16.56	0.45	0.42	**6.94**	**2.60**	**0.24**	**36.75**	**−20.34**	**0.80**
	Meadow type (patchy)	16.56	0.49	0.49	**6.94**	**0.12**	**0.33**	**36.75**	**−4.47**	**0.95**
	Meadow type (variable)	16.56	−0.19	0.47	**6.94**	**−0.89**	**0.37**	**36.75**	**−1.69**	**1.08**

Significant terms (p-value < 0.05) are shown in bold. ∆AIC represents the AIC difference between the non-spatial GLS and the spatial GLS accounting for spatial autocorrelation on the response variable; positive value means lower AIC on the spatial GLS model. SE, standard error. (*) shows continuous explanatory variables.

**Figure 1:**
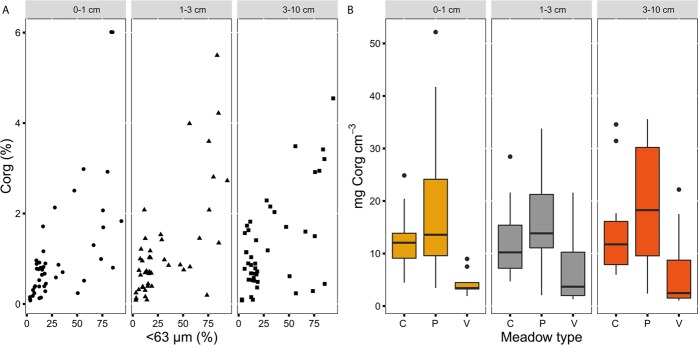
Relationships among sediment carbon variables and drivers per each depth core section. (**a**) Biplot of C_org_ content (%) and sediment grain size <63 µm (%); (**b**) Boxplot of C_org_ stocks (mg C_org_ cm^−3^) and meadow type. Symbols for grain size: circle, depth section 0–1 cm; triangle, depth section 1–3 cm; quadrat, depth section 3–10 cm. Legend for meadow type: C, permanent continuous meadows; P, permanent patchy meadows; V, variable meadows.

**Figure 2:**
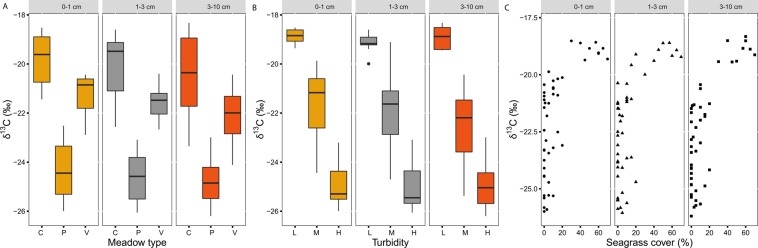
Relationships among sediment carbon sources and drivers per each depth core section. (**a**) Boxplot of δ^13^C (‰) and meadow type; (**b**) Boxplot of δ^13^C (‰) and turbidity; (**c**) δ^13^C (‰) and seagrass cover. Legend for meadow type: C, permanent continuous meadows; P, permanent patchy meadows; V, variable meadows. Legend for water turbidity: H, high; M, medium; L, low. Symbols for seagrass cover: circle, depth section 0–1 cm; triangle, depth section 1–3 cm; quadrat, depth section 3–10 cm.

The C_org_ (%) content in the sediment for all core sections was positively related with the proportion of fine fraction of sediments (Fig. 1a, p ≤ 0.02, Table 2). The C_org_ stocks (mg C_org_ cm^−3^) in the sediment for all core sections were significantly related to meadow type (p ≤ 0.02, Table 2). The highest C_org_ stocks were associated with continuous meadow types and patchy meadows, although there was a high variability present in patchy meadows as shown by the model estimates’ standard errors and confidence intervals (Supplementary Table S3, Fig. 1b). The δ^13^C values in the sediments for all core sections were significantly related to meadow type, turbidity and seagrass cover (p ≤ 0.01, Table 2). The most depleted values were associated with patchy meadows (Fig. 2a) and to high water turbidity (Fig. 2b); and these were positively related with seagrass cover (Fig. 2c).

**Figure 3:**
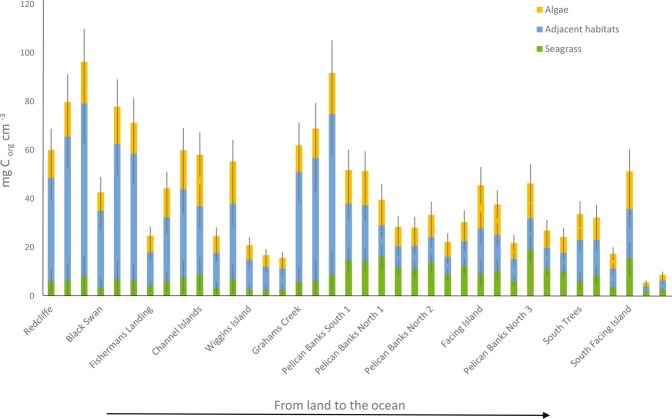
Contribution of the different carbon sources to total sediment C_org_ stocks (mg C_org_ cm^−3^) of seagrass meadows on each core within the estuary. Sources: adjacent habitats sources (including mangroves and saltmarshes); marine algae (including benthic algae and seston); seagrass sources. This figure is based on the mean results and standard deviation of each source fraction from mixing models (see Table 3). Total sediment C_org_ stocks (mg C_org_ cm^−3^) represent the cumulative amount of C_org_ on each 10 cm depth core.

The mixing models applied indicated that allochthonous, non-seagrass, sources were the most important source of sediment C_org_ (contribution range from 58% to 92%; Table 3), followed by seagrass (contribution range from 8% to 42%; Table 3). In general, seagrass presented a minor contribution to sedimentary C_org_ stocks along the estuary (~8 mg C_org_ cm^−3^) (Fig. 3), although this contribution increased in the lower regions of the estuary (up to ~20 mg C_org_ cm^−3^), coincident with the presence of the largest seagrass meadows in the estuary (Table 1). The contribution from adjacent habitat sources and marine algae was higher (~35 mg C_org_ cm^−3^ on average) and also decreasing along a gradient from the upper (with up to ~92 mg C_org_ cm^−3^) to the lower parts of the estuary (Fig. 3).

**Table 3:** Results from the mixing models.

	Adjacent habitats (mangroves & saltmarshes)	Algae (benthic algae & seston)	Seagrass	Allochthonous sources	Autochthonous sources
Mean	50.57	26.65	22.79	77.21	22.79
SD	15.50	5.50	12.37	12.37	12.37
Min	27.45	17.59	8.12	58.39	8.12
Max	74.07	39.04	41.61	91.88	41.61

Values represent summary statistics of the proportion (%) of each source (mean, standard deviation and range by minimum and maximum values). Autochthonous sources represent the seagrass contribution, while allochthonous sources represent the sum of non-seagrass sources.

**Figure 4:**
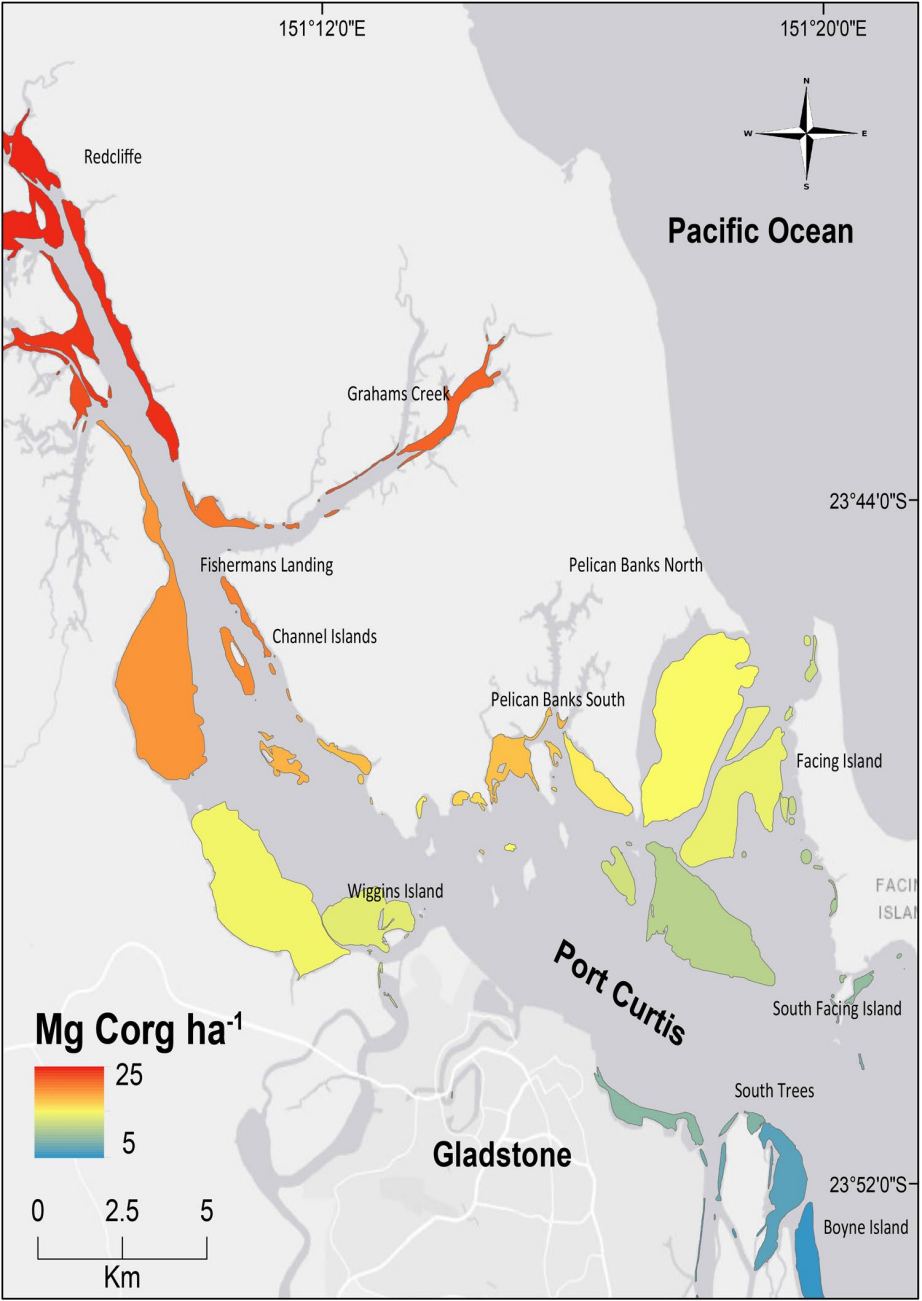
Predictive map of C_org_ stocks (Mg C_org_ ha^−1^) in the first 10 cm of sediment of seagrass meadows within the Port Curtis estuary, Queensland, Australia, based on data from this study. The map was built using Esri ArcGIS 10.4 (www.esri.com/software/arcgis).

Predicted seagrass C_org_ stocks within the estuary were five times higher in the upper regions than in the lower regions, with C_org_ stocks ranging from 25 Mg C_org_ ha^−1^ to 5 Mg C_org_ ha^−1^ in the first 10 cm of sediment (Fig. 4).

## Discussion

The distribution of sedimentary C_org_ storage in seagrass meadows within the Port Curtis estuary, Queensland, Australia, was spatially heterogeneous, following a general pattern of higher C_org_ accumulation in sediments of seagrass meadows in the upper reaches of the estuary than in seagrass meadows at the estuary mouth. Contrary to our expectations, seagrass sedimentary C_org_ content (%) or C_org_ stocks (mg C_org_ cm^−3^) did not increase with seagrass cover. Although, C_org_ content (%) was significantly related with a higher proportion of fine sediments and sedimentary C_org_ stocks (mg C_org_ cm^−3^) were related with the meadow type. Water turbidity was related with carbon sources, as well as seagrass cover and meadow type. Thus, a higher seagrass contribution was found in sedimentary C_org_ in continuous meadows, which also presented higher seagrass cover and low water turbidity. Despite this, the contribution from seagrasses (as a source) to C_org_ stored in sediments was still very low through all seagrass meadows in the estuary. Overall, sedimentary C_org_ storage in seagrass meadows within the estuary studied seems to be governed by the accumulation of fine sediments and allochthonous carbon sources along a gradient from land to the ocean, suggesting that these patterns should be considered when quantifying C_org_ stocks at similar scales.

Sedimentary C_org_ beneath seagrass meadows in the upper parts of the estuary, were more than ten times higher than in the lower estuary. The highest values in the upper estuary are comparable to those from long-lived seagrass species of the *Posidonia* genus, while values for the same seagrass species (*Zostera muelleri*) in the lower estuary are comparable to other fast-growing seagrass species^7^ including those from estuaries within the same region^14,37^. The strong spatial gradient on sedimentary C_org_ stocks in seagrass meadows exerted from the upper to the lower estuary suggests that the estuary studied is governed by a strong hydrodynamic control of carbon deposition processes^28^. Therefore, it is likely that a higher deposition of fine sediments, carrying particulate organic materials through freshwater inputs (e.g. land runoff), is occurring in the upper parts of the estuary. This explains the higher carbon levels in these seagrass meadows and is corroborated also by the more depleted values of δ^13^C in these areas^38^. These results highlight the necessity to account for spatial gradients when assessing C_org_ stocks within estuaries to adequately account for the variability present. Our results also support that spatial autocorrelation is an important consideration when assessing drivers of C_org_ stocks where strong spatial gradients are present^20^.

Among the main environmental and biological factors explaining the variability found within the estuary, the proportion of fine sediments appeared as the only significant explanatory variable for C_org_ content (%) in sediments. Seagrass sediment carbon storage has been widely related with a high proportion of fine sediments^12,39^ as fine sediments will slow down decomposition processes by avoiding oxygenation^40^. In agreement with our results, recent studies suggest that in fast growing seagrass species, such as the ones in this study, this relationship is even stronger when the contribution of seagrass-derived carbon to the sedimentary C_org_ pool is relatively low^41^.

Seagrass meadow type (i.e. landscape configuration) was among the main factors associated with sedimentary C_org_ stocks (mg C_org_ cm^−3^) and carbon sources within the estuary. As shown by the spatial models applied C_org_ stocks were positively related with continuous seagrass meadows and negatively related with variable non-permanent meadows, while results on patchy meadows were uncertain due to the high variability present. In fact, in the different patchy meadows sampled in this study, sedimentary C_org_ storage varied by one order of magnitude depending on the location within the estuary. In addition, the cores with the highest records of C_org_ stocks on this study were from patchy meadows in the upper extreme of the estuary, demonstrating that the spatial location of seagrass meadows within the estuary matters in Blue Carbon assessments. On the other hand, continuous meadows showed a low variability in sedimentary C_org_ stocks, and a higher contribution from seagrass sources. This could be related to higher, and probably more constant, burial of seagrass-derived carbon in continuous meadows, compared to patchy and non-permanent variable meadows, promoting more accumulation and burial of seagrass-derived material^18^, that are generally less labile and will decompose at lower rates promoting long lasting C_org_ stocks^42^. This relationship could also be related with the capacity of seagrass meadows to avoid resuspension and mixing of sediments^36,43–45^, which is presumably higher in continuous meadows^18^. This fact is also corroborated by the positive relationship found here among seagrass cover and carbon isotopic signature, and the low isotopic signature of C_org_ in patchy and non-permanent variable seagrass meadows suggesting less seagrass contribution in these meadows.

Although we found a significant relationship between carbon sources and water turbidity, but not water turbidity and C_org_ stocks (mg C_org_ cm^−3^), our results support the hypothesis^14^ that high sediment carbon storage occurs in areas of high turbidity due to high levels of allochthonous carbon. High water turbidity was related with depleted values of δ^13^C, suggesting a high contribution of allochthonous sources, while low water turbidity was related with δ^13^C values close to seagrass sources. The latter suggests two potential mechanisms: (1) in low turbidity conditions, higher levels of light promote seagrass growth, and so, more seagrass contribution to sediment C_org_; and (2) that the seagrass canopies filtering capacity decreases water turbidity, thus relating seagrass contribution and low turbidity (see also results for seagrass cover and meadow type). In fact, low water turbidity occurred only in areas with presence of continuous seagrass meadows. However, in this study we did not assess the relative importance of each driver compared to the others, making it difficult to make conclusions about the particular processes involved, which should be further studied. In addition, if measured during the rainy season, water turbidity could have led to different results in the surface sediments, by affecting seagrass physiology (e.g. photosynthetic processes), sedimentation and eventually C_org_ storage. Finally, despite the presence of mangroves being highlighted as an important factor promoting seagrass carbon storage in other studies^35^, our results showed no relationship between proximity to mangroves and seagrass sedimentary C_org_ for any variable assessed, probably due to the low carbon exchange between those habitats within the estuary.

These results suggest that, within this estuary, the seagrass plant itself is not governing the amount of C_org_ stored in the sediment, and that multiple other interacting factors are involved (e.g. sediment deposition). Light availability is known to be a major control of seagrass growth^46^ and seems to be dominating seagrass meadows distribution within the estuary. The upper parts of the estuary presented higher water turbidity, a low seagrass cover, and a higher presence of patchy meadows when compared with the lower parts of the estuary, usually with less turbid water, more seagrass cover and where continuous meadows are found. Although seagrass cover is usually associated with higher C_org_ storage^12^, in this estuary, seagrass cover values found were small, and seagrass sedimentary C_org_ seems overwhelmingly driven by the proximity to the upper estuary. This leads to an inverse correlation between C_org_ and seagrass cover that we suggest is coincidental and driven by the light environment rather than a causal link between seagrass cover and low C_org_ storage.

In this study plant traits other than seagrass cover were not measured. However, annual seagrass monitoring in Port Curtis estuary since 2009 show that aboveground biomass follows the same spatial pattern as seagrass cover in the same study sites used here, ranging from 0.88 g DW m^−2^ in the upper estuary to 18.02 g DW m^−2^ in continuous meadows at the estuary mouth^47–49^. All seagrass species found in the estuary are considered fast growing, characterized by a small size and a small above- and below-ground fraction, with roots and rhizomes that do not penetrate deeper than the first few centimetres. This could be related with the small seagrass contribution to carbon storage found in this estuary. Small intra-specific or inter-specific variation in plant traits could potentially influence spatial patterns of carbon storage^14,20^ and should be included in further studies.

Although seagrass cover was a determinant of the type of carbon sources entering into the sediments, the contribution of seagrasses as carbon sources along the estuary was low. The increase of seagrass contribution to sedimentary C_org_ in the lower parts of the estuary could be related to a lower deposition of allochthonous carbon (compared to the upper parts), and also a major presence of continuous meadows that could facilitate the trapping and burial of seagrass materials into the sediments and a higher local production contributing autochthonous carbon^50^. Despite this, allochthonous sources were the most important contributors, and followed the same spatial pattern as C_org_ stocks within the estuary.

Overall, fine sediments and allochthonous carbon sources accumulating more in the upper parts of the estuary seem to be driving spatial heterogeneity found in sedimentary C_org_ storage in seagrass meadows within the estuary studied. This highlights the necessity to account for the river-ocean continuum gradients on Blue Carbon assessments within-estuaries. We therefore recommend, when assessing carbon stocks in seagrass meadows within estuaries, to identify the main sources of variation, and, in order to adequately capture the highest variability, sample sediments in different seagrass meadows following the main spatial gradients. Comprehensive and accurate assessments of sediment C_org_ stocks are required to understand the role of estuaries as carbon sinks, and ultimately disentangle their total carbon budget and climate change mitigation potential.

## Materials and methods

### Study site and sampling design

The study was conducted in Port Curtis (23°46′57″S; 151°18′0″E), a macro-tidal estuary on the central Queensland coast of north-eastern Australia. Port Curtis is a large natural harbour that has been industrialized and urbanized over the last half century. Despite this, much of it remains in a relatively natural state with a forested catchment dominating, and mangrove vegetation followed by saltmarshes landward of the mangroves in the upper estuary. The estuary contains large intertidal and subtidal sand and mud flats, which support seagrass meadows dominated mainly by the species *Zostera muelleri*, with *Halodule uninervis, Halophila decipiens, Halophila ovalis* and *Halophila spinulosa* also present. Seagrass appears sparsely distributed in the upper reaches of the estuary while meadows become larger (and more continuous) on the lower parts^47–49^. These relatively low cover seagrass meadows are typical of much of the tropical and sub-tropical Queensland coast where they play key roles in supporting megagrazers, such as dugong and green turtles, and as a fish habitat for juvenile commercial and recreationally important species^51^.

**Figure 5:**
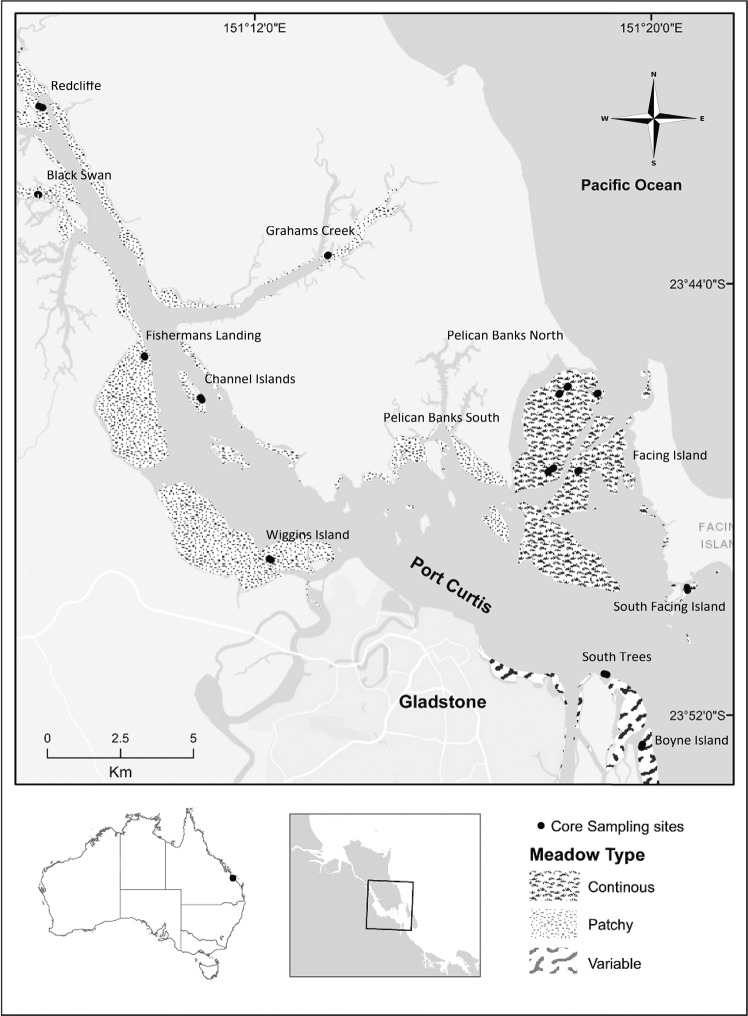
Map of the Port Curtis estuary, Queensland, Australia, and areas of seagrass distribution. Sediment core locations are indicated with black dots. The map was built using Esri ArcGIS 10.4 (www.esri.com/software/arcgis).

To study the C_org_ storage variability of seagrass sediments within the estuary, and to identify the main factors promoting it, we sampled across all areas of seagrass distribution within the estuary (Fig. 5)^47,48^. We sampled a total of 45 sediment cores in order to cover the main environmental and biological gradients within the estuary. Thus, sediment cores were taken in 15 sites along the estuary in groups of three. In each site, cores were sampled with a minimum distance of 50 m among them and sampling all the seagrass species present inside each site in order to embrace all potential variability present (Table 1). Cores of sediments were sampled at low tide by manually inserting open-barrel PVC pipes (20 cm length, 5 cm internal diameter) into soils to a depth of 10 cm and using a piston to provide suction as cores were withdrawn. Compaction during coring was low (<10%). Once extracted, cores were capped at both ends and transported to the laboratory. Cores were kept upright during transport to prevent mixing of sediment layers within the core. GPS coordinates and a 50 × 50 cm photo quadrat were taken at each core location. Seagrass cover (%) and species composition in each quadrat were estimated visually following Seagrass-Watch percent cover standards^52^.

### Laboratory procedures

In the laboratory, the sediments were sliced into three sections at 0–1, 1–3, 3–10 cm intervals. Coarse inorganic particles (i.e. large carbonate materials) and living plant material were manually removed^7,9^. Depth sediment sections were dried at 60°C and weighed in order to calculate bulk density. Each depth section was then homogenized by mixing the sediments with a clean stainless-steel spoon thoroughly or until visually homogeneous, and split into two sub-samples, with grain size particle distribution analysed from the first subsample using a Malvern Mastersizer 2000 laser microgranulometer. Prior to grain size analysis, organic matter in this sub-sample was removed by addition of hydrogen peroxide 10%. Particle size distribution was expressed as percentage (%) of volume for particle diameters from 0 to 2000 µm. The second sub-sample was ground to a fine powder with a laboratory ball grinding mill and split again into two sub-samples for C_org_ and N elemental and isotopic analysis. Half were washed with acid for C_org_ analysis, and the other half remained untreated, as this chemical procedure has been reported to alter δ^15^N values^53^. Sub-samples were acidified drop by drop with HCl 1M, until there was no visual evidence of effervescence to remove any carbonates and re-dried without rinsing^54,55^. After drying, samples were re-ground, placed in tin capsules and analysed for C_org_ and N elemental and isotopic composition. Measurements of C_org_ and N elemental composition and stable isotope ratios were performed using a continuous-flow isotope-ratio mass spectrometer MAT253 (Thermo Finnigan) coupled to an elemental analyser EA1108 (Carlo Erba Instruments) through a Conflo III interface (ThermoFinnigan). The C and N isotope ratios are expressed as δ values in parts per thousand (‰) relative to Vienna Pee Dee Belemnite and the atmospheric air standard, respectively, according to standard notation (δX = [*(R*sample/*R*standard) − 1] × 1000, where *R* is the ratio ^13^C/^12^C or ^15^N/^14^N). International Atomic Energy Agency standards were inserted every 12 samples for calibration. Replicate assays of standards indicated measurement errors of ± 0.1 and ± 0.2 ‰ for C and N, respectively. Standing C_org_ stocks per volume unit were calculated using dry bulk density data and C_org_ content and expressed as mg C_org_ cm^−3^.

### Environmental and biological drivers of seagrass carbon storage

Environmental and biological drivers of seagrass carbon storage were characterized using categorical and numerical variables as a function of the seagrass cover, meadow type, sediment particle size, water turbidity and their proximity to other vegetated habitats such as mangroves (Table 1). We did not compare among the different seagrass species due to the low number of cores for some of them (Table 1) and cores from different species were integrated in data analysis (see below). Meadow type was classified based on definitions by^47,49^ using three categories, permanent continuous meadows, permanent patchy meadows and variable meadows, the last defining non-permanent meadows that have been reported to appear intermittently as continuous or as isolated or aggregated patches. The proportion (%) of the smallest sediment fraction (silt and clay < 63 µm) was used for further statistical analysis. Water turbidity was classified using three categories, based on monthly averaged data from Gladstone Ports Corporation^56^ in Nephelometric Turbidity Units (NTU) measured in the bottom of the water column during the dry season (May to October) where: low was <6 NTU, medium <7–15>, high 15 NTU>. Finally, we calculated the shortest distance from each core to mangroves using aerial photographs (Google Earth, 2013) and Euclidean distance measures representing shortest travel paths (ArcGIS 10.4; ESRI Software Inc).

### Data analysis

Due to the potential presence of spatial gradients within the estuary and the grouped sampling of the sediment cores, we initially estimated the spatial autocorrelation in carbon sediment variables C_org_ (%), δ^13^C (‰) and C_org_ stocks (mg C_org_ cm^−3^) for each core depth section by calculating Moran I statistics. We then used generalized least squares (GLS) to model carbon sediment variables for each core depth in relation to the environmental and biological explanatory variables (i.e. *seagrass cover (%), meadow type, <63 µm (%), distance to mangroves (m)* and *turbidity)*. Each explanatory variable was assessed individually in separate models given the high correlation among them. All GLS models were developed with and without spatial correlation structure in the error term of the regression model (GLS spatial models and GLS non-spatial models, respectively) to model spatially autocorrelated residuals when present^57^. Exponential, rational quadratic, gaussian, linear and spherical correlation structures were assessed for each spatial GLS model. The best fitting model and correlation structure were defined by the minimum Akaike’s Information Criterion (AIC)^58–61^. The non-spatial GLS model is equivalent to Ordinary Least Squares (OLS), and the spatial GLS extends OLS by providing for possibly unequal residual variances and covariance of residuals between locations^62^. Finally, we tested for spatially autocorrelated residuals visualizing semivariograms of normalized residuals and calculating Moran’s I statistic for the GLS non-spatial and GLS spatial models. Normality and homoscedasticity were explored in final models via visual estimation of trends of model residuals (errors associated with homogeneity of variance, independence, and normality). We also checked if the 95% confidence intervals for parameter estimates of numerical explanatory variables were reasonable or included zero, which indicates fitting problems^63^. We fit all the models using restricted maximum likelihood with the nlme package^64^, and Moran tests and semivariograms were done with package ape^65^ and spdep^66^ in the R Statistical Computing Environment. Non-transformed values (means ± SE) are shown in the figures and tables. For all analyses, core section values of C_org_ (%) and C_org_ stocks (mg C_org_ cm^−3^) were log transformed.

The Bayesian mixing model SIAR 4.2^67^ in the R Statistical Computing Environment was used to estimate the contribution of potential sources to the sedimentary C_org_ pool. The model was run with two isotopes (δ^13^C and δ^15^N) and three sources (see Supplementary Fig. S1): seagrasses (δ^13^C −12.94 ‰ ± 3.08 SD; δ^15^N 4.06 ‰ ± 1.31 SD), adjacent habitat sources (including mangroves and saltmarshes) (δ^13^C −26.23 ‰ ± 1.03 SD; δ^15^N 3.4 ‰ ± 1.1 SD), and marine algae (including benthic algae and seston) (δ^13^C −21.34 ‰ ± 3.29 SD; δ^15^N 4.69 ‰ ± 1.47 SD). Isotopic signatures of sources were collected and averaged from previous studies in the same area (see Supplementary Table S4). Separate mixing models were computed for each core. The isotopic values for all sources were assumed to be constant for each model. We did not consider any fractionation with aging (0 ‰) in the model because previous studies suggest small diagenetic shifts for δ^13^C and δ^15^N during decomposition^68,69^. The proportion of each source on the mixing model outputs was used to calculate the total contribution of each source to the C_org_ stocks accumulated per core (the sum of mg C_org_ cm^−3^ in 10 cm of core depth).

Finally, to elaborate a predictive map of C_org_ stocks, and their variability, at the estuary scale the total C_org_ stock in the first 10 cm soil depth in tonnes per hectare (Mg C_org_ha^−1^) was interpolated across known areas of seagrass distribution^47–49^ in the estuary extent using ArcGIS and the Empirical Bayesian kriging tool Geostatistical Analyst extension (ArcGIS 10.4; ESRI Software Inc). Areas of seagrass distribution integrated information of all seagrass species within the estuary because of the non-monospecific nature of most seagrass meadows. Empirical Bayesian kriging is an interpolation method that accounts for the error in estimating the underlying semivariogram through repeated simulations^70^. The standard deviation from each site (see Table 1) was interpolated to provide a measure of variability in the values and used as error terms. For each seagrass defined area or seagrass patch we calculated the mean and error term per hectare.

## Supplementary information

Supplementary information.

## Data Availability

The datasets generated during and/or analysed during the current study are available from the corresponding author on reasonable request.
