# Supplementary material for: High variability of Blue Carbon storage in seagrass meadows at the estuary scale
Source: Sci Rep. 2020 Apr 3;10:5865. doi: 10.1038/s41598-020-62639-y (PMC7125166; doi:10.1038/s41598-020-62639-y)
Supplement: Supplementary file 1 — Supplementary information. [file 41598_2020_62639_MOESM1_ESM.pdf]

Supplementary Materials:

High variability of blue carbon storage in seagrass meadows at the estuary scale

Aurora M Ricart[1,2*], Paul H. York[3], Catherine V. Bryant[3], Michael A. Rasheed[3], Daniel Ierodiaconou[4] & Peter I. Macreadie[4]

[1] *Bodega Marine Laboratory- Department of Earth and Planetary Sciences- University of California Davis, USA.*

[2] *Departament de Biologia Evolutiva, Ecologia i Ciències ambientals, Universitat de Barcelona, Spain.*

[3] *Centre for Tropical Water and Aquatic Ecosystem Research, James Cook University, Australia*

[4] *School of Life and Environmental Sciences, Centre for Integrative Ecology, Deakin University, Australia*

**corresponding author; email amricart@ucdavis.edu, auroramartinez@ub.edu*

Fig S1. Isotope biplot of $\delta^{13}C$ and $\delta^{15}N$ in parts per thousand (‰) for sediment samples (dots) and sources used on mixing models. Sources show mean and standard deviation. Symbols: triangle, adjacent habitats sources (including mangroves and saltmarshes); circle, marine algae (including benthic algae and seston); quadrat, seagrass.

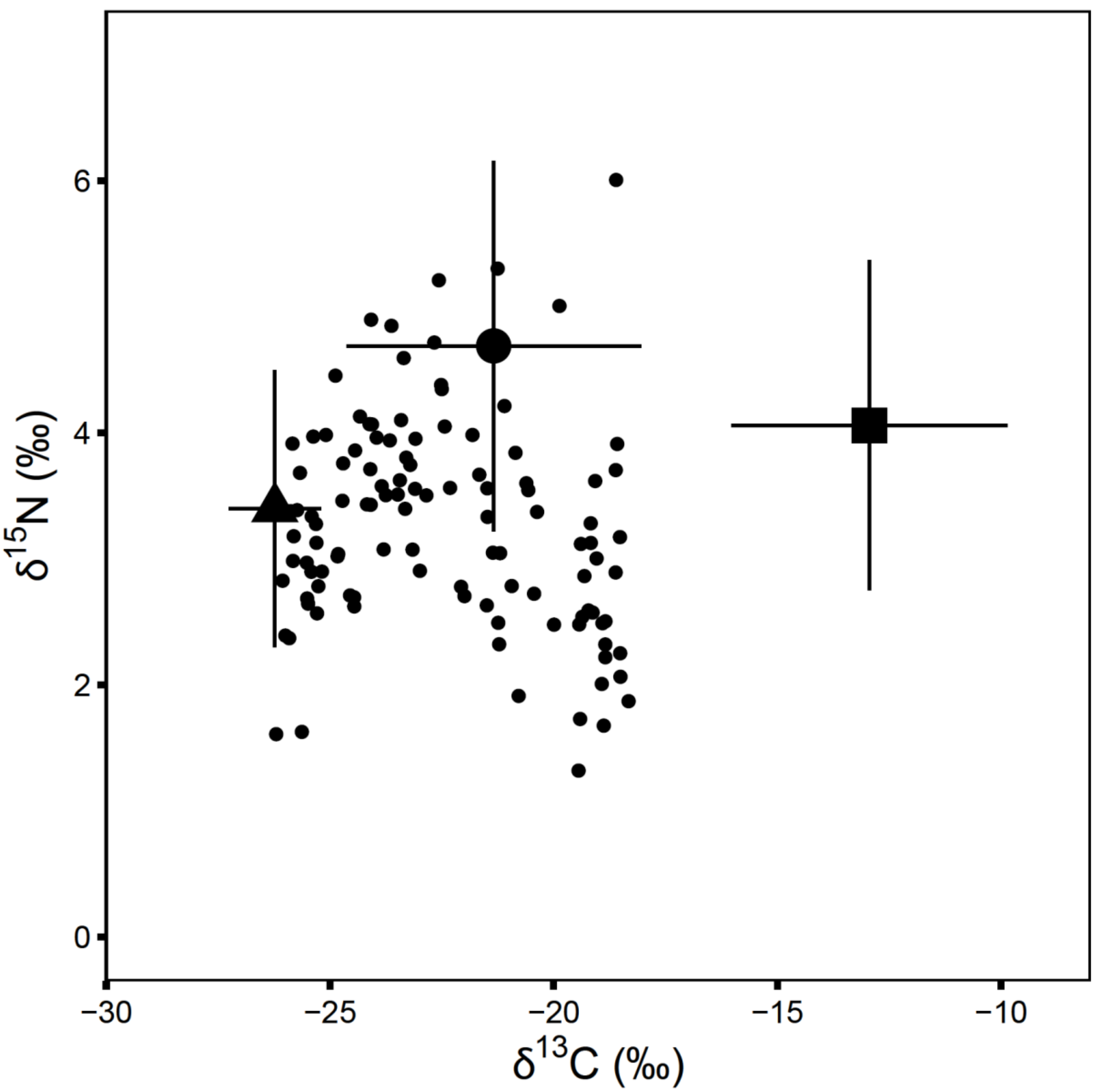

Table S1. Results of Moran's I test for sediment carbon variables from the original data

Sediment carbon variable	Depth	Moran I			
		Observed	Expected	SD	p-value
C_{org} (%)	0-1cm	-0.23	-0.02	0.02	<0.01
	1-3 cm	-0.21	-0.02	0.02	<0.01
	3-10cm	-0.20	-0.02	0.02	<0.01
C stocks (mgC_{org} cm^{-3})	0-1cm	-0.20	-0.02	0.02	<0.01
	1-3 cm	-0.11	-0.02	0.02	<0.01
	3-10cm	-0.10	-0.02	0.02	<0.01
$\delta^{13}C$ (‰)	0-1cm	-0.39	-0.02	0.02	<0.01
	1-3 cm	-0.38	-0.02	0.02	<0.01
	3-10cm	-0.34	-0.02	0.02	<0.01

Table S2. Results of Moran's I test for residual spatial autocorrelation in non-spatial and spatial GLS models of carbon sediment variables. Significant terms (p-value <0.05) are shown in bold. SD, standard deviation. (*) shows continuous explanatory variables

Dependent variable	Depth	Driver	Non-spatial Moran I				Spatial Moran I			
			Observed	Expected	SD	p-value	Observed	Expected	SD	p-value
C_{org} (%)	0-1cm	Seagrass cover	-0.21	-0.02	0.02	<0.01	-0.01	-0.02	0.02	**0.59**
		<63µm*	-0.06	-0.02	0.02	0.02	-0.02	-0.02	0.02	**0.80**
		Distance from mangroves*	-0.17	-0.02	0.02	<0.01	-0.01	-0.02	0.02	**0.56**
		Turbidity	-0.08	-0.02	0.02	<0.01	-0.01	-0.02	0.02	**0.50**
		Meadow type	-0.05	-0.02	0.02	**0.10**	-0.02	-0.02	0.02	**0.65**
C_{org} (%)	1-3 cm	Seagrass cover	-0.18	-0.02	0.02	<0.01	-0.02	-0.02	0.02	**0.75**
		<63µm*	-0.04	-0.02	0.02	**0.31**	-0.01	-0.02	0.02	**0.78**
		Distance from mangroves*	-0.15	-0.02	0.02	<0.01	-0.03	-0.02	0.02	**0.66**
		Turbidity	-0.07	-0.02	0.02	0.01	-0.01	-0.02	0.02	**0.76**
		Meadow type	-0.04	-0.02	0.02	**0.41**	-0.02	-0.02	0.02	**0.67**
C_{org} (%)	3-10cm	Seagrass cover	-0.17	-0.02	0.02	<0.01	-0.01	-0.02	0.02	**0.52**
		<63µm*	-0.05	-0.02	0.02	**0.06**	-0.02	-0.02	0.02	**0.95**
		Distance from mangroves*	-0.15	-0.02	0.02	<0.01	-0.01	-0.02	0.02	**0.57**
		Turbidity	-0.08	-0.02	0.02	0.00	-0.01	-0.02	0.02	**0.40**
		Meadow type	-0.04	-0.02	0.02	**0.22**	-0.02	-0.02	0.02	**0.78**
Cstocks (mgC$_{org}$ cm^{-3})	0-1cm	Seagrass cover	-0.20	-0.02	0.02	<0.01	-0.02	-0.02	0.02	**0.85**
		<63µm*	-0.09	-0.02	0.02	<0.01	-0.02	-0.02	0.02	**0.97**
		Distance from mangroves*	-0.19	-0.02	0.02	<0.01	-0.03	-0.02	0.02	**0.72**
		Turbidity	-0.10	-0.02	0.02	<0.01	-0.02	-0.02	0.02	**0.71**
		Meadow type	-0.05	-0.02	0.02	**0.16**	-0.02	-0.02	0.02	**0.83**
Cstocks (mgC$_{org}$ cm^{-3})	1-3 cm	Seagrass cover	-0.11	-0.02	0.02	<0.01	-0.04	-0.02	0.02	**0.26**
		<63µm*	-0.04	-0.02	0.02	**0.15**	-0.02	-0.02	0.02	**0.92**
		Distance from mangroves*	-0.11	-0.02	0.02	<0.01	-0.04	-0.02	0.02	**0.24**
		Turbidity	-0.06	-0.02	0.02	<0.05	0.02	-0.02	0.02	**1.00**
		Meadow type	-0.02	-0.02	0.02	**0.92**	-0.01	-0.02	0.02	**0.40**
Cstocks (mgC$_{org}$ cm^{-3})	3-10cm	Seagrass cover	-0.10	-0.02	0.02	<0.01	-0.02	-0.02	0.02	**0.98**
		<63µm*	-0.06	-0.02	0.02	0.03	-0.02	-0.02	0.02	**0.96**
		Distance from mangroves*	-0.12	-0.02	0.02	<0.01	-0.04	-0.02	0.02	**0.46**
		Turbidity	-0.07	-0.02	0.02	<0.01	-0.02	-0.02	0.02	**0.66**
		Meadow type	-0.02	-0.02	0.02	**0.95**	-0.01	-0.02	0.02	**0.43**
$\delta^{13}C$ (‰)	0-1cm	Seagrass cover	-0.35	-0.02	0.02	<0.01	-0.03	-0.02	0.02	**0.73**
		<63µm*	-0.14	-0.02	0.02	<0.01	-0.03	-0.02	0.02	**0.74**
		Distance from mangroves*	-0.22	-0.02	0.02	<0.01	-0.04	-0.02	0.02	**0.34**
		Turbidity	-0.17	-0.02	0.02	<0.01	-0.02	-0.02	0.02	**0.86**
		Meadow type	-0.04	-0.02	0.02	**0.42**	-0.01	-0.02	0.02	**0.34**
$\delta^{13}C$ (‰)	1-3 cm	Seagrass cover	-0.34	-0.02	0.02	<0.01	-0.05	-0.02	0.02	**0.10**
		<63µm*	-0.14	-0.02	0.02	<0.01	-0.09	-0.02	0.02	<0.01
		Distance from mangroves*	-0.21	-0.02	0.02	<0.01	-0.08	-0.02	0.02	<0.01
		Turbidity	-0.16	-0.02	0.02	<0.01	-0.07	-0.02	0.02	<0.01
		Meadow type	-0.03	-0.02	0.02	**0.65**	-0.01	-0.02	0.02	**0.32**
$\delta^{13}C$ (‰)	3-10cm	Seagrass cover	-0.30	-0.02	0.02	<0.01	-0.01	-0.02	0.02	**0.45**
		<63µm*	-0.12	-0.02	0.02	<0.01	-0.03	-0.02	0.02	**0.72**
		Distance from mangroves*	-0.19	-0.02	0.02	<0.01	-0.03	-0.02	0.02	**0.76**
		Turbidity	-0.15	-0.02	0.02	<0.01	-0.02	-0.02	0.02	**0.92**
		Meadow type	-0.03	-0.02	0.02	**0.61**	-0.01	-0.02	0.02	**0.30**

Table S3. Coefficient estimates for carbon sediment variables in GLS spatial models. Significant drivers (p-value <0.05) are shown in bold. ΔAIC represents the AIC difference between the non-spatial GLS and the spatial GLS accounting for spatial autocorrelation on the response variable; positive value means lower AIC on the spatial GLS model. Semivariogram distances are based on latitude and longitude decimal degrees. The correlation structured used on the final model is specified. df, degrees of freedom. SE, standard error. CI 95%, confidence interval. (*) shows continuous explanatory variables

Dependent variable	Depth	Driver	Semivariogram		ΔAIC	Correlation structure	df	p-value	Estimate	SE	CI 95%	
			Range	Nugget							lower 95%	upper 95%
C_{org} (%)	0-1cm	seagrass cover*	0.05	0.13	38.75	Ratio	45 (43 res)	0.53	0.00	0.00	-0.01	0.01
		**<63μm***	**0.06**	**0.23**	**12.28**	**Ratio**	**45 (43 res)**	**0.02**	**0.01**	**0.00**	**0.00**	**0.01**
		distance to mangroves*	0.05	0.12	36.74	Ratio	45 (43 res)	0.97	0.00	0.00	0.00	0.00
		turbidity (low)	0.06	0.14	22.78	Gaussian	45 (42 res)	0.17	0.55	0.34	-0.13	1.23
		turbidity (medium)	0.06	0.14	22.78	Gaussian	45 (42 res)	0.17	-0.20	0.27	-0.73	0.32
		turbidity (high)	0.06	0.14	22.78	Gaussian	45 (42 res)	0.17	0.42	0.39	-0.34	1.17
		meadow type (continuous)	0.05	0.13	24.38	Gaussian	45 (42 res)	0.47	0.51	0.47	-0.42	1.42
		meadow type (patchy)	0.05	0.13	24.38	Gaussian	45 (42 res)	0.47	0.42	0.55	-0.65	1.48
		meadow type (variable)	0.05	0.13	24.38	Gaussian	45 (42 res)	0.47	-0.19	0.56	-1.29	0.91
C_{org} (%)	1-3 cm	seagrass cover*	0.06	0.16	28.51	Ratio	45 (43 res)	0.44	0.00	0.00	-0.01	0.00
		**<63μm***	**0.04**	**0.29**	**8.06**	**Ratio**	**45 (43 res)**	**0.02**	**0.01**	**0.00**	**0.00**	**0.01**
		distance to mangroves*	0.05	0.15	30.54	Ratio	45 (43 res)	0.72	0.00	0.00	0.00	0.00
		turbidity (low)	0.05	0.19	17.52	Gaussian	45 (42 res)	0.18	0.34	0.32	-0.29	0.97
		turbidity (medium)	0.05	0.19	17.52	Gaussian	45 (42 res)	0.18	0.12	0.27	-0.41	0.65
		turbidity (high)	0.05	0.19	17.52	Gaussian	45 (42 res)	0.18	0.67	0.38	-0.07	1.41
		meadow type (patchy)	0.05	0.18	19.21	Gaussian	45 (42 res)	0.31	0.46	0.43	-0.39	1.31
		meadow type (continuous)	0.05	0.18	19.21	Gaussian	45 (42 res)	0.31	0.50	0.50	-0.48	1.48
		meadow type (variable)	0.05	0.18	19.21	Gaussian	45 (42 res)	0.31	-0.19	0.52	-1.21	0.83
C_{org} (%)	3-10cm	seagrass cover*	1.41	0.04	26.84	Linear	45 (43 res)	0.12	-0.01	0.00	-0.01	0.00
		**<63μm***	**0.00**	**0.16**	**8.94**	**Ratio**	**45 (43 res)**	**0.01**	**0.01**	**0.00**	**0.00**	**0.01**
		distance to mangroves*	1.76	0.02	27.19	Linear	45 (43 res)	0.93	0.00	0.00	0.00	0.00
		turbidity (low)	0.05	0.24	16.28	Gaussian	45 (42 res)	0.31	0.38	0.31	-0.23	0.99
		turbidity (medium)	0.05	0.24	16.28	Gaussian	45 (42 res)	0.31	0.19	0.27	-0.33	0.71
		turbidity (high)	0.05	0.24	16.28	Gaussian	45 (42 res)	0.31	0.56	0.37	-0.15	1.28
		meadow type (patchy)	0.06	0.21	16.56	Gaussian	45 (42 res)	0.35	0.45	0.42	-0.38	1.27
		meadow type (continuous)	0.06	0.21	16.56	Gaussian	45 (42 res)	0.35	0.49	0.49	-0.47	1.45
		meadow type (variable)	0.06	0.21	16.56	Gaussian	45 (42 res)	0.35	-0.19	0.47	-1.11	0.73
Cstocks (mgC_{org} cm^{-3})	0-1cm	seagrass cover*	0.18	0.11	30.61	Ratio	45 (43 res)	0.53	0.00	0.00	-0.01	0.01
		<63μm*	0.17	0.14	18.44	Ratio	45 (43 res)	0.17	0.00	0.00	0.00	0.01
		distance to mangroves*	0.01	0.00	33.64	Ratio	45 (43 res)	0.18	0.00	0.00	0.00	0.00
		turbidity (low)	0.11	0.17	19.26	Ratio	45 (42 res)	0.67	2.23	0.64	0.99	3.48
		turbidity (medium)	0.11	0.17	19.26	Ratio	45 (42 res)	0.67	-0.12	0.29	-0.68	0.44
		turbidity (high)	0.11	0.17	19.26	Ratio	45 (42 res)	0.67	0.16	0.40	-0.62	0.93
		**meadow type (continuous)**	**0.00**	**0.27**	**5.34**	**Ratio**	**45 (42 res)**	**<0.01**	**2.52**	**0.17**	**2.19**	**2.86**
		**meadow type (patchy)**	**0.00**	**0.27**	**5.34**	**Ratio**	**45 (42 res)**	**<0.01**	**0.20**	**0.24**	**-0.26**	**0.66**
		**meadow type (variable)**	**0.00**	**0.27**	**5.34**	**Ratio**	**45 (42 res)**	**<0.01**	**-0.90**	**0.27**	**-1.44**	**-0.37**

Cstocks ($mgC_{org}\ cm^{-3}$)	1-3 cm	seagrass cover*	0.00	0.18	7.64	Linear	45 (43 res)	0.77	0.00	0.01	-0.01	0.01
		<63µm*	0.00	0.37	3.21	Gaus	45 (43 res)	0.12	0.01	0.00	0.00	0.02
		distance to mangroves*	0.00	0.27	8.57	Gaus	45 (43 res)	0.99	0.00	0.00	0.00	0.00
		turbidity (low)	0.64	0.11	9.27	Linear	45 (42 res)	0.51	1.61	1.29	-0.92	4.16
		turbidity (medium)	0.64	0.11	9.27	Linear	45 (42 res)	0.51	0.44	0.40	-0.34	1.23
		turbidity (high)	0.64	0.11	9.27	Linear	45 (42 res)	0.51	0.50	0.51	-0.50	1.51
		**meadow type (continuous)**	**0.00**	**0.29**	**2.87**	**Gaus**	**45 (42 res)**	**0.02**	**2.47**	**0.20**	**2.08**	**2.87**
		**meadow type (patchy)**	**0.00**	**0.29**	**2.87**	**Gaus**	**45 (42 res)**	**0.02**	**0.21**	**0.27**	**-0.33**	**0.75**
		**meadow type (variable)**	**0.00**	**0.29**	**2.87**	**Gaus**	**45 (42 res)**	**0.02**	**-0.65**	**0.31**	**-1.26**	**-0.04**
Cstocks ($mgC_{org}\ cm^{-3}$)	3-10cm	seagrass cover*	5.69	0.01	13.19	Linear	45 (43 res)	0.22	-0.01	0.01	-0.02	0.01
		<63µm*	0.00	0.07	9.92	Ratio	45 (43 res)	0.32	0.00	0.00	0.00	0.01
		distance to mangroves*	0.00	0.08	11.85	Ratio	45 (43 res)	0.77	0.00	0.00	0.00	0.00
		turbidity (low)	0.59	0.09	13.04	Linear	45 (42 res)	0.41	1.62	1.53	-1.38	4.62
		turbidity (medium)	0.59	0.09	13.04	Linear	45 (42 res)	0.41	0.63	0.48	-0.31	1.57
		turbidity (high)	0.59	0.09	13.04	Linear	45 (42 res)	0.41	0.38	0.61	-0.82	1.60
		**meadow type (continuous)**	**0.00**	**0.11**	**6.94**	**Gaus**	**45 (42 res)**	**0.02**	**2.60**	**0.24**	**2.13**	**3.08**
		**meadow type (patchy)**	**0.00**	**0.11**	**6.94**	**Gaus**	**45 (42 res)**	**0.02**	**0.12**	**0.33**	**-0.52**	**0.78**
		**meadow type (variable)**	**0.00**	**0.11**	**6.94**	**Gaus**	**45 (42 res)**	**0.02**	**-0.89**	**0.37**	**-1.62**	**-0.17**
$\delta^{13}C$ (‰)	0-1cm	**seagrass cover***	**0.31**	**0.06**	**63.93**	**Linear**	**45 (43 res)**	**0.01**	**0.03**	**0.01**	**0.01**	**0.05**
		<63um*	0.07	0.08	47.21	Ratio	45 (43 res)	0.23	-0.01	0.01	-0.03	0.01
		distance to mangroves*	0.09	0.05	71.45	Ratio	45 (43 res)	0.16	0.00	0.00	0.00	0.00
		**turbidity (low)**	**0.11**	**0.17**	**36.68**	**Gaussian**	**45 (42 res)**	**<0.01**	**-20.14**	**0.83**	**-21.76**	**-18.51**
		**turbidity (medium)**	**0.11**	**0.17**	**36.68**	**Gaussian**	**45 (42 res)**	**<0.01**	**-2.09**	**0.42**	**-2.91**	**-1.27**
		**turbidity (high)**	**0.11**	**0.17**	**36.68**	**Gaussian**	**45 (42 res)**	**<0.01**	**-4.05**	**0.57**	**-5.17**	**-2.93**
		**meadow type (continuous)**	**0.04**	**0.24**	**24.64**	**Gaussian**	**45 (42 res)**	**<0.01**	**-19.94**	**0.96**	**-21.82**	**-18.06**
		**meadow type (patchy)**	**0.04**	**0.24**	**24.64**	**Gaussian**	**45 (42 res)**	**<0.01**	**-4.55**	**1.11**	**-6.71**	**-2.38**
		**meadow type (variable)**	**0.04**	**0.24**	**24.64**	**Gaussian**	**45 (42 res)**	**<0.01**	**-1.12**	**1.24**	**-3.55**	**1.29**
$\delta^{13}C$ (‰)	1-3 cm	**seagrass cover***	**8.15**	**0.00**	**61.09**	**Linear**	**45 (43 res)**	**<0.01**	**0.04**	**0.01**	**0.01**	**0.06**
		<63µm*	0.07	0.07	48.06	Ratio	45 (43 res)	0.64	0.00	0.01	-0.02	0.01
		distance to mangroves*	0.01	0.08	60.62	Ratio	45 (43 res)	0.07	0.00	0.00	0.00	0.00
		**turbidity (low)**	**0.00**	**0.18**	**29.26**	**Gaussian**	**45 (42 res)**	**<0.01**	**-19.11**	**0.76**	**-20.59**	**-17.62**
		**turbidity (medium)**	**0.00**	**0.18**	**29.26**	**Gaussian**	**45 (42 res)**	**<0.01**	**-2.95**	**0.87**	**-4.67**	**-1.24**
		**turbidity (high)**	**0.00**	**0.18**	**29.26**	**Gaussian**	**45 (42 res)**	**<0.01**	**-5.93**	**0.95**	**-7.80**	**-4.05**
		**meadow type (continuous)**	**0.00**	**0.23**	**21.41**	**Gaussian**	**45 (42 res)**	**<0.01**	**-19.95**	**0.46**	**-20.85**	**-19.04**
		**meadow type (patchy)**	**0.00**	**0.23**	**21.41**	**Gaussian**	**45 (42 res)**	**<0.01**	**-4.71**	**0.62**	**-5.92**	**-3.49**
		**meadow type (variable)**	**0.00**	**0.23**	**21.41**	**Gaussian**	**45 (42 res)**	**<0.01**	**-1.67**	**0.74**	**-3.12**	**-0.22**
$\delta^{13}C$ (‰)	3-10cm	**seagrass cover***	**0.97**	**0.01**	**48.87**	**Exponential**	**45 (43 res)**	**<0.01**	**0.04**	**0.01**	**0.02**	**0.06**
		<63µm*	0.03	0.07	55.64	Ratio	45 (43 res)	0.61	0.00	0.01	-0.02	0.01
		distance to mangroves*	0.03	0.07	69.80	Ratio	45 (43 res)	0.52	0.00	0.00	0.00	0.00
		**turbidity (low)**	**0.02**	**0.18**	**31.19**	**Gaussian**	**45 (42 res)**	**<0.01**	**-18.97**	**1.10**	**-21.14**	**-16.80**
		**turbidity (medium)**	**0.02**	**0.18**	**31.19**	**Gaussian**	**45 (42 res)**	**<0.01**	**-3.74**	**1.15**	**-5.99**	**-1.49**
		**turbidity (high)**	**0.02**	**0.18**	**31.19**	**Gaussian**	**45 (42 res)**	**<0.01**	**-5.95**	**1.29**	**-8.49**	**-3.42**

		**meadow type (continuous)**	**0.02**	**0.20**	**36.75**	**Gaussian**	**45 (42 res)**	**<0.01**	**-20.34**	**0.80**	**-21.92**	**-18.76**
		**meadow type (patchy)**	**0.02**	**0.20**	**36.75**	**Gaussian**	**45 (42 res)**	**<0.01**	**-4.47**	**0.95**	**-6.34**	**-2.60**
		**meadow type (variable)**	**0.02**	**0.20**	**36.75**	**Gaussian**	**45 (42 res)**	**<0.01**	**-1.69**	**1.08**	**-3.81**	**0.42**

Table S4. Sources stable isotopes data from other studies in the area

**δ¹⁵N**	**Sources**															
	Mangrove			Saltmarsh		Seagrass			Algae							Seston
Literature reference	*Rhizosphora stylosa*	*Avicennia marina*	*Aegiceras corniculatum*	Grass	Succulents	*Zostera muelleri*	*Halophila ovalis*	*Halodule uninervis*	*Catenella nipae*	*Spyridia filamentosa*	*Chondria sp.*	*Laurencia sp.*	*Sargassum sp.*	*Dyctiota ligulata*	Green filamentous	
From Connolly and others 2006				2	4		*5*									5
From Melzer & Johnson 2004	4.77	4.20	3.80			3.82							3.78			
From Melzer & Johnson 2004	3.22	3.42	1.21			3.43							3.92			
From Melzer & Johnson 2004	4.02	5.60				5.13										
From Melzer & Johnson 2004	1.86	5.36				3.23										
From Melzer & Johnson 2004	3.45	3.52				3.23										
From Melzer & Johnson 2004	3.50	2.75														
From Melzer & Johnson 2004	3.88	3.93														
From Melzer & Johnson 2004	3.34															
From Melzer & Johnson 2004	4.34															
From Prior and others 2015	3.70	2.20	3.70				5.30	2.10	3.20	2.90	2.50	3.40	3.10	7.50	3.80	
From Loneragan and others 1997	1.90						3.10	3.00								5.30
From Loneragan and others 1997	3.40						6.50	3.30								5.70
From Loneragan and others 1997	3.70							5.70								5.90
From Loneragan and others 1997	1.30															5.40
From Loneragan and others 1997																5.70
From Loneragan and others 1997																6.60
From Loneragan and others 1997																6.10

$\delta^{13}C$	Sources																
	Mangrove			Saltmarsh		Seagrass			Algae							Seston
Literature reference	*Rhizosphora stylosa*	*Avicennia marina*	*Aegiceras corniculatum*	Grass	Succulents	*Zostera* spp.	*Halophila ovalis*	*Halodule uninervis*	*Catenella nipae*	*Spyridia filamentosa*	Chondria sp.	*Laurencia* spp.	*Sargassum* spp.	*Dyctiota ligulata*	Green filamentous	
From Connolly and others 2006				-26	-15	-14	-19									-22
From Prior and others 2015	-27.4	-27.2	-26.5			-8.6	-9.6	-11.7	-28.6	-27.8	-19	-18.2	-15.6	-21.6	-22.1	
From Loneragan and others 1997	-28.7						-10.7	-12.1								-22
From Loneragan and others 1997	-28.3						-15.4	-15								-21.7
From Loneragan and others 1997	-28.2							-13.3								-19.3
From Loneragan and others 1997	-28.8															-23.2
From Loneragan and others 1997																-19.1
From Loneragan and others 1997																-21.9
From Loneragan and others 1997																-19.3
From Loneragan and others 1997																-20.1

References used in Table S4

Connolly RM, Melville AJ, Hindell JS, Preston KM. 2003. Whole estuary analysis of the contribution of estuarine autotrophs to fish and crustaceans over unvegetated mudflats in Port Curtis. In: Stable isotope tracing of the contribution of seagrass production to subtropical fisheries species occurring outside seagrass areas. Griffith University. p26-36.

Loneragan NR, Bunn SE, Kellaway DM. 1997. Are mangroves and seagrasses sources of organic carbon for penaeid prawns in a tropical Australian estuary? A multiple stable-isotope study. Marine Biology 130: 289-300.

Melzer A, Johnson R. 2004. Stable isotopes of nitrogen as potential indicators of nitrogen contamination in Port Curtis: A Pilot Study. Stable isotopes in Curtis Coast mangroves. A report to Coastal CRC, Centre for Environmental Management & Cooperative Research Centre for Coastal Zone, Estuary and Waterway Management.

Prior B, Booth DT, Limpus CJ. 2016. Investigating diet and diet switching in green turtles (*Chelonia mydas*). Australian Journal of Zoology 63: 365-375.
